# Distinct Driver Pathway Enrichments and a High Prevalence of *TSC2* Mutations in Right Colon Cancer in Chile: A Preliminary Comparative Analysis

**DOI:** 10.3390/ijms25094695

**Published:** 2024-04-25

**Authors:** Camilo Tapia-Valladares, Guillermo Valenzuela, Evelin González, Ignacio Maureira, Jessica Toro, Matías Freire, Gonzalo Sepúlveda-Hermosilla, Diego Ampuero, Alejandro Blanco, Iván Gallegos, Fernanda Morales, José I. Erices, Olga Barajas, Mónica Ahumada, Héctor R. Contreras, Jaime González, Ricardo Armisén, Katherine Marcelain

**Affiliations:** 1 Departamento de Oncología Básico Clínico, Facultad de Medicina, Universidad de Chile, Santiago 8380000, Chile; 2 Centro de Genética y Genómica, Instituto de Ciencias e Innovación en Medicina, Facultad de Medicina Clínica Alemana, Universidad del Desarrollo, Santiago 7610507, Chile; 3 Departamento de Tecnología Médica, Facultad de Medicina, Universidad de Chile, Santiago 8380000, Chile; 4 Centro para la Prevención y Control del Cáncer, CECAN, Universidad de Chile, Santiago 8380000, Chile; 5 CORFO Center of Excellence in Precision Medicine, Pfizer Chile, Santiago 8380000, Chile; 6 Departamento de Patología, Hospital Clínico de la Universidad de Chile, Santiago 8380453, Chile; 7 Departamento de Medicina Interna, Hospital Clínico de la Universidad de Chile, Santiago 8380453, Chile

**Keywords:** Colorectal cancer, TSC2, LATAM, NGS, precision medicine

## Abstract

Colorectal cancer (CRC) is the second leading cause of cancer deaths globally. While ethnic differences in driver gene mutations have been documented, the South American population remains understudied at the genomic level, despite facing a rising burden of CRC. We analyzed tumors of 40 Chilean CRC patients (Chp) using next-generation sequencing and compared them to data from mainly Caucasian cohorts (TCGA and MSK-IMPACT). We identified 388 mutations in 96 out of 135 genes, with *TP53* (45%), *KRAS* (30%), *PIK3CA* (22.5%), *ATM* (20%), and *POLE* (20%) being the most frequently mutated. *TSC2* mutations were associated with right colon cancer (44.44% in RCRC vs. 6.45% in LCRC, *p*-value = 0.016), and overall frequency was higher compared to TCGA (*p*-value = 1.847 × 10^−5^) and MSK-IMPACT cohorts (*p*-value = 3.062 × 10^−2^). Limited sample size restricts definitive conclusions, but our data suggest potential differences in driver mutations for Chilean patients, being that the RTK-RAS oncogenic pathway is less affected and the PI3K pathway is more altered in Chp compared to TCGA (45% vs. 25.56%, respectively). The prevalence of actionable pathways and driver mutations can guide therapeutic choices, but can also impact treatment effectiveness. Thus, these findings warrant further investigation in larger Chilean cohorts to confirm these initial observations. Understanding population-specific driver mutations can guide the development of precision medicine programs for CRC patients.

## 1. Introduction

Colorectal cancer (CRC) is the third most common type of cancer in incidence and the second in mortality worldwide, with incidence rates rising among South American countries over the last few years [1]. Among the risk factors are lifestyle behaviors such as diet, physical activity, obesity, alcohol consumption, and tobacco use [2].

CRC originates from the epithelial cells of the colon and rectum through a progressive and sequential accumulation of genetic alterations that primarily transform a normal epithelium into an adenoma and finally into a carcinoma [3]. The mechanisms leading to the production of these genetic alterations can be divided into three different pathways: chromosomal instability (CIN), microsatellite instability (MSI), and CpG island methylator phenotype (CIMP) [4].

CRC can develop throughout the colon and rectum, being more frequent in the distal or left colon, which is comprised of the colon from the splenic flexure up to the rectum. In contrast, the right or proximal colon is the segment including the caecum, ascending colon, and transverse colon. Right colon cancer (RCRC) is more associated with mucinous histology, *BRAF* mutation, and the MSI pathway, whereas in left colon cancer (LCRC), amplification of *EGFR* and *ERBB2*, *TP53* mutation, and CIN pathway are more common [5,6,7,8].

Although the mutational landscape in CRC is well known, differences in the frequency of mutations related to ethnicity have been reported; a higher frequency of mutations in *PIK3CA*, *MAP2K1*, and *NF1* have been seen in tumor samples from patients of African American ethnicity compared to Caucasians [9]. Conversely, a higher frequency of mutations in *BRAF* has been reported in tumor samples of patients of Caucasian ethnicity when compared with samples from Asian and African American subjects [10] and between the Western and Chinese populations [11].

Anti-EGFR targeted therapy has become the primary treatment option for patients with advanced disease. The presence of mutations in downstream effectors *KRAS*, *NRAS*, and *BRAF* are proven to confer resistance to the treatments with anti-EGFR agents [12,13,14]. Nevertheless, an important proportion of patients have poor or no response to this therapy despite having no detectable mutations in these genes [15,16]. Over the last years, mutations in other genes, such as *PIK3CA* and *PTEN*, have been proposed to predict response to anti-EGFR therapy [16,17,18,19,20]. However, these associations remain to be further investigated.

Thus, differences in the tumor mutational landscape between populations may translate into a distinct population-specific prevalence of driver mutations and actionable pathways and, after that, in a particular therapy program.

Numerous studies have been conducted to get a deeper insight into mutations and target therapy association in CRC. However, the Latin American population is often underrepresented in them. This entails, among other things, a need for knowledge about the real benefit this population might get from current and new targeted therapies, or which one better fits this population’s requirements.

This pilot study offers a unique contribution to colorectal cancer research by analyzing cohort of 40 Chilean patients. The admixed genomes of modern Chileans reflect ancestral contributions primarily from Europe and Native America, with a minor African influence. The Native American component originates from two major indigenous groups: the Mapuche from southern Chile, and the Aymara and Quechua populations of the north. Among European influences, Chileans exhibit greater genetic similarity to Spaniards and Italians compared to British or CEU (Utah Residents with Northern and Western European Ancestry) populations.

Given the limited data available on Latin American populations in colorectal cancer research, we focused on a Chilean cohort to gain further insights into the potential for population-specific genomic characteristics.

Comparisons with established cohorts like MSK-IMPACT and TCGA suggest potential differences in mutation frequencies, particularly in the PI3K pathway, where the Chilean cohort appears to have a higher frequency. While the limited sample size restricts definitive conclusions, these initial observations highlight the importance of including more diverse populations in colorectal cancer research. Understanding population-specific variations in driver mutations can inform the development of personalized therapeutic strategies for a wider range of patients.

## 2. Results

### 2.1. Clinical and General Characteristics of the Patients

In this study, 40 primary tumors were assessed. In total, 23 of them were from males, and 17 were from females. The median age at the diagnosis was 65.5 (ranging from 25 to 86) years. The histological diagnoses were tubular adenocarcinoma in 45% (18/40) of the cases, adenocarcinoma not otherwise specified (NOS) in 32.5% (13/40), 5% (2/40) papillary tubular adenocarcinomas, 2.5% (1/40) poorly differentiated with signet-cells carcinoma, 2.5% (1/40) adenocarcinoma with mixed mucinous and tubular subtypes, one mucinous adenocarcinoma (2.5%, 1/40), and 10% (4/40) not specified. CRC was mainly found in the left colon (LCRC), with thirty-one tumors, whereas nine were in the right colon (RCRC) (*p*-value = 1.508 × 10^−6^). Patients with LCRC were predominantly males (twenty males vs. eleven females), whereas patients with RCRC were predominantly females (six females vs. three males), but without a statistically significant difference. In addition, we explored the difference in CRC diagnosis based on the self-reported ethnicity of patients; however, we found no difference in the occurrence of LCRC and RCRC depending on ethnicity (Table 1).

### 2.2. General Landscape

**Figure 1:**
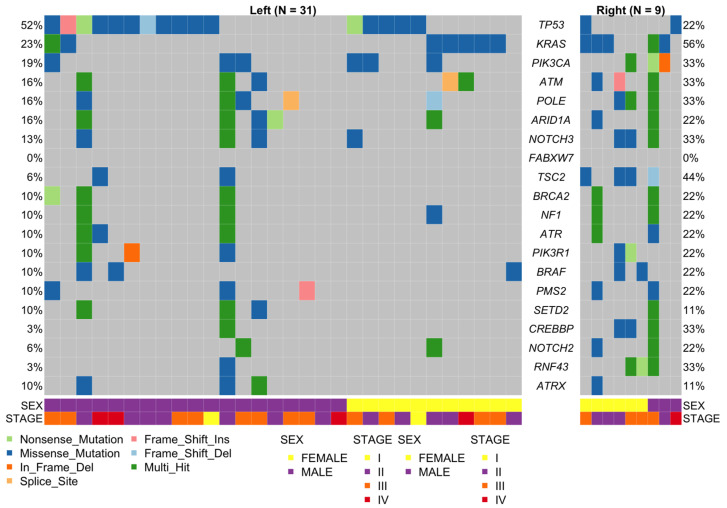
Distribution and mutation type for the 20 most mutated genes found in 37 out of 40 tumor samples, separated by sidedness. The lower panel shows the sex and stage distributions. Mutation types are depicted in a color code.

We detected 388 somatic non-synonymous single-nucleotide variants (SNV) and Indels in 96 out of 135 genes. The most frequent type of mutations were missense mutations (79.12%), followed by nonsense mutations (14.69%). The most frequently mutated genes were TP53 (45%), KRAS (30%), PIK3CA (22.5%), ATM (20%), and POLE (20%) (Figure 1). The median for the number of somatic variants was 3, the lower quartile (25%) was 1, whereas the higher quartile (75%) was 5.75 (ranging from 0 to 81). One patient had no detectable somatic variants (male, tumor of rectum, age at diagnosis 79 years), and there were three hypermutated tumors with 64, 73, and 81 somatic variants. These three individuals were all males, 66, 42, and 52 years old at diagnosis: the first two with left colon cancer and the last with right colon cancer. Unfortunately, no information about MSI status was available for these tumors.

One female patient was very young at diagnosis (25 years, left colon cancer), with MSI high, assessed using IHC (loss of expression of MLH1 and PMS2) and PCR. This patient had no family history of cancer, BRAF mutations, MLH1, PMS2, MSH2, or MSH6 mutations, but, interestingly, had mutations in POLE (c.2091delC) and BAP1 (c.G160A).

### 2.3. Mutational Status of Actionable Genes in CRC

#### 2.3.1. KRAS, NRAS, and BRAF

Determination of the mutation status of *KRAS*, *NRAS*, and *BRAF* is the primary recommendation for patients being considered for anti-EGFR therapy in two major guidelines [21,22]. Fourteen *KRAS* mutations were detected in 30% (12/40) of the samples and were concentrated in exon 2, codons 12 and 13 (11 mutations), with two patients having two mutations. The remaining mutations were in exon 2, codon 23, and two in exon 3, codons 59 and 92. On the other hand, only one sample (2.5%) had a mutation *NRAS* in exon 3, codon 60 (Figure 1 and Supplementary Table S1).

Finally, 12.5% (5/40) of the patients had mutations detected in *BRAF*. These were concentrated in exon 15, three corresponding to V600E hotspot mutation, while the remaining two were in exon 11 (codon 469) and exon 15 (codon 584).

#### 2.3.2. MLH1, PMS2, MSH2, and MSH6

Mutations in the genes coding for the mismatch repair proteins have been related to mismatch repair deficiency (dMMR) [23], which is a condition that needs to be evaluated for patients being considered for checkpoint inhibitor therapies [24]. Seven patients had mutations in *PMS2*, *MSH2*, and/or *MSH6*, and no mutations were found in *MLH1*. The patients (six males and one female) had an average age at diagnosis of 60.57 (±11.96, ranging from 42 to 75). Among the men, there was only one RCRC, whereas the only woman had also a RCRC. Five mutations in five patients were seen in *PMS2*, four missense mutations, and one frameshift insertion. Six mutations in *MSH2* were found in four samples. three of them in exon 11, and one in exons 4, 10, and 15. Two were non-previously reported mutations (c.G680T and c.A2495G). Finally, five mutations were found in 7.5% (3/40) of the patients in MSH6. Unfortunately, MSI/MSS statuses were not available for these patients (Figure 1 and Supplementary Table S1).

### 2.4. Mutational Status of Emerging Predictive and Actionable Genes

#### 2.4.1. PIK3CA

Although there is no current clinical recommendation for PIK3CA testing in CRC samples, mutations in this gene could confer resistance to anti-EGFR therapies and, on the other side, represent an opportunity for therapy [25,26]. Ten mutations were found in 22.5% of the patients (9/40); most of them were in exon 10 (four mutations), followed by exon 2 (three mutations). Two mutations were found in exon 21 and one was found in exon 5. One of the mutations in exon 2 was a novel in-frame deletion (c.335_337del). Other mutated genes in the PI3K-AKT-mTOR pathway were *TSC1* (two mutations in two samples, 5%), *TSC2* (six mutations in six samples, 15%, Figure 1), and *PTEN* (six mutations in four samples, 10%). Notably, one patient had three mutations in *PTEN*, including a non-previously reported mutation in exon 1 (c.G21T). The remainder were all in exons 5 and 8 (three and two, respectively). Finally, four mutations in four samples (10%) were detected in *MTOR* (Supplementary Table S1).

#### 2.4.2. TP53

Mutations in *TP53* have been related to reduced sensitivity to anti-EGFR therapy [27]. Mutations in this gene were found in 45% (18/40) of the patients. Most of them were missense mutations followed by frameshift and nonsense mutations (14, 2, and 2, respectively). Eight were in exon 1, two were in exon 2, three were in exon 3, four were in exon 4, and one was in exon 6.

### 2.5. Left Versus Right-Sided CRC

**Figure 2:**
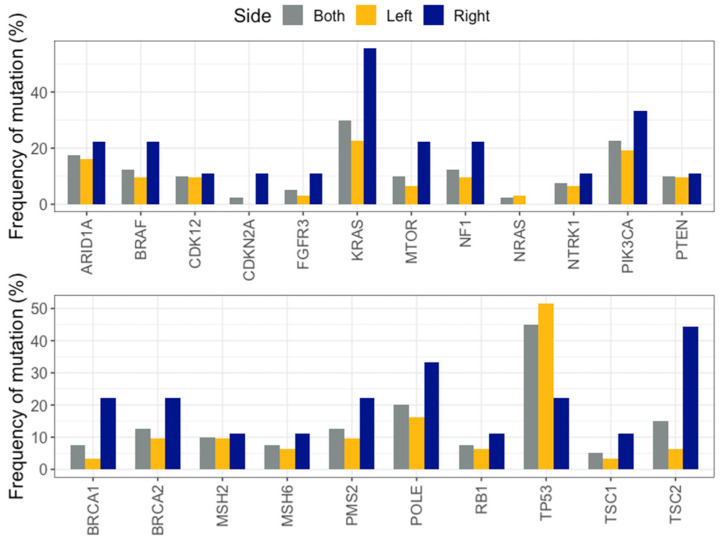
Mutation frequencies for clinically relevant genes (**upper panel**) and other genes of interest (**lower panel**) in LCRC, RCRC, and both sides.

**Figure 3:**
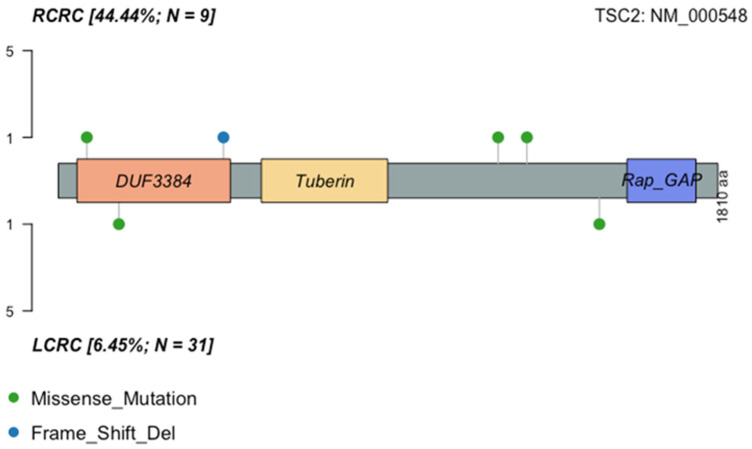
Distribution of mutations in TSC2 protein in the Chp cohort. Amino acid changes are displayed by sidedness.

Out of the 40 samples sequenced, 31 corresponded to LCRC samples and 9 to RCRC. The mean age at diagnosis for LCRC was 61.35 (range 25–82) years and for RCRC was 71.22 (range 51–86) years, without a statistically significant difference (Wilcoxon test, *p*-value = 0.062). Several genes were found to tend to be mutated in one location or the other. TP53 was found to be mutated predominantly in LCRC, whereas *KRAS*, *BRAF*, and *PIK3CA* were found to be mainly in RCRC. The only mutation detected in *NRAS* was found in an LCRC sample. Additionally, diverse genes related to DNA damage repair mechanisms tended to be more frequently mutated in RCRC than in LCRC (*BRCA1*, *BRCA2*, *MSH6*, *PMS2*, and *POLE*). However, the only gene with a significant difference was *TSC2* (44.44% in RCRC vs. 6.45% in LCRC, *p*-value = 0.016) (Figure 2 and Figure 3).

### 2.6. Comparison of Mutation Frequencies in Independent Cohorts

**Figure 4:**
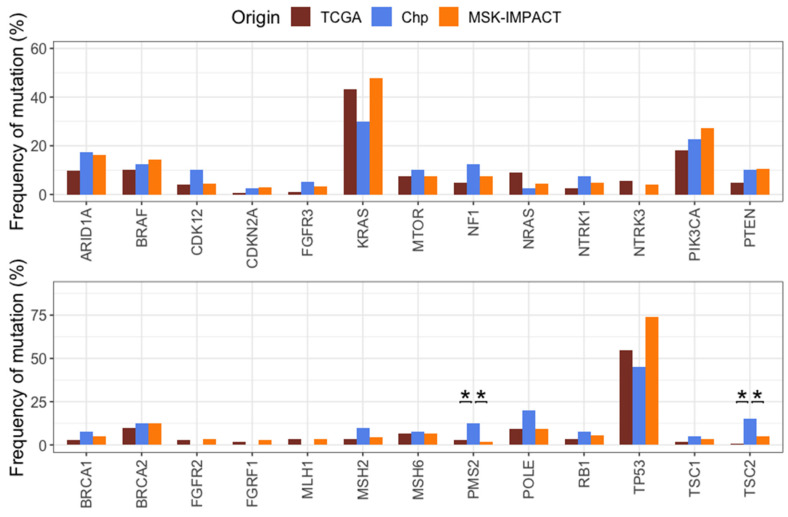
Mutation frequencies of clinically relevant genes (**upper panel**) and other genes of interest (**lower panel**) in TCGA, MSK-IMPACT, and Chp patients. Only statistically significant differences between both groups compared to Chp cohort are indicated (* *p* < 0.05, chi-square test).

To determine differences or similarities among our cohort (Chilean patients, Chp) and other populations, colorectal cancer data from TCGA and MSK-IMPACT databases was obtained through cBioportal. The OncoKB database for colorectal cancer was consulted for the selection of genes with clinical implications, and mutation frequencies on selected genes were compared among the three cohorts. Despite the limited sample size, significant differences were found for *NRAS*, (*p* = 3.563 × 10^−2^), *PIK3CA* (*p* = 2.153 × 10^−2^), *PTEN* (0.04155), *PMS2* (*p* = 8.651 × 10^−5^), *TP53* (*p* = 1.698 × 10^−6^), and *TSC2* (*p* = 1.463 × 10^−4^). All the remaining comparisons did not indicate statistically significant differences when compared all the three groups at once (Supplementary Table S2). Afterward, pairwise comparisons were made to determine if the difference for every gene was between TCGA vs. Chp or MSK-IMPACT vs. Chp. Significant differences, both for TCGA vs. Chp for *TSC2* (*p* = 1.847 × 10^−5^) and *PMS2* (*p* = 1.532 × 10^−2^), and MSK-IMPACT vs. Chp for *TSC2* (*p* = 3.062 × 10^−2^) and for *PMS2* (*p* = 1.125 × 10^−4^), were found (Figure 4). *NRAS*, *PIK3CA*, and *TP53* were significant when comparing MSK-IMPACT vs. TCGA (*p*-values = 2.64 × 10^−2^, 7.87 × 10^−3^ and 5.74 × 10^−7^, respectively), but not when comparing to Chp (*p*-values = 0.85, 0.62, and 0.15 for MSK-IMPACT vs. Chp, and *p*-values = 0.28, 0.64, and 0.73 for TCGA vs. Chp, respectively). *PIK3R1* and *PTEN* showed a higher frequency of mutations in Chp compared to TCGA (15% vs. 5.38%, *p*-value = 0.06 and 15% vs. 4.93%, *p*-value = 0.042) (Figure 4).

### 2.7. Left Versus Right Sided CRC in MSK-IMPACT and TCGA Cohorts

LCRC and RCRC were compared independently in both the MSK-IMPACT and the TCGA cohorts. Except for *TP53*, all the remaining statistically significant differences were related to genes more frequently mutated in RCRC when compared to LCRC in both cohorts. Common to both cohorts were the differences found in *BRAF*, *PIK3CA*, and *TP53*, whereas statistically significant differences were found in *ARID1A*, *BRCA2*, *CDK12*, *FGFR3*, *KRAS*, *MTOR*, *NF1*, *NTRK1*, *NTRK3*, *MSH2*, *MSH6*, *POLE*, *PTEN*, *RB1*, *TSC1*, and *TSC2* only in the MSK-IMPACT cohort, although with a tendency for agreement with the TCGA cohort, with exception of *TSC2*, which has no mutated cases in the RCRC cases (Table 2).

### 2.8. Pathway Analyses

**Figure 5:**
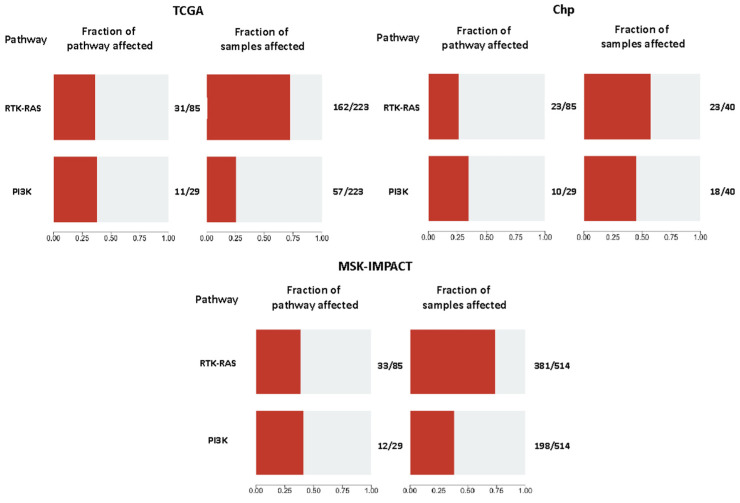
Fraction of the mutated genes considered in each pathway (**on the left**) and the fraction of the samples affected by those mutations (**on the right**) in every cohort.

To determine if there were any other differences not being detected by comparing individual genes, a general comparison based on mutated genes of the RTK-RAS and PI3K pathways among cohorts was made using the OncogenicPathways tool from the Maftools package. A significantly higher fraction of samples with altered PI3K pathway was found in the Chp and the MSK-IMPACT cohorts when compared to TCGA (45%, 38.52%, and 25.56%, respectively, *p*-values = 0.02 for Chp vs. TCGA, and 0.000921 for MSK-IMPACT vs. TCGA). Overall, there was not a significant difference as a group in the RTK-RAS pathway. However, a statistically significant difference was found between Chp and MSK-IMPACT in a pairwise comparison (57.5% vs. 74.12%, *p*-value = 0.03622), whereas for Chp vs. TCGA, the *p*-value was not low enough, but a tendency was observed (57.5% vs. 72.65%, respectively, *p*-value = 0.08) (Figure 5).

**Figure 6:**
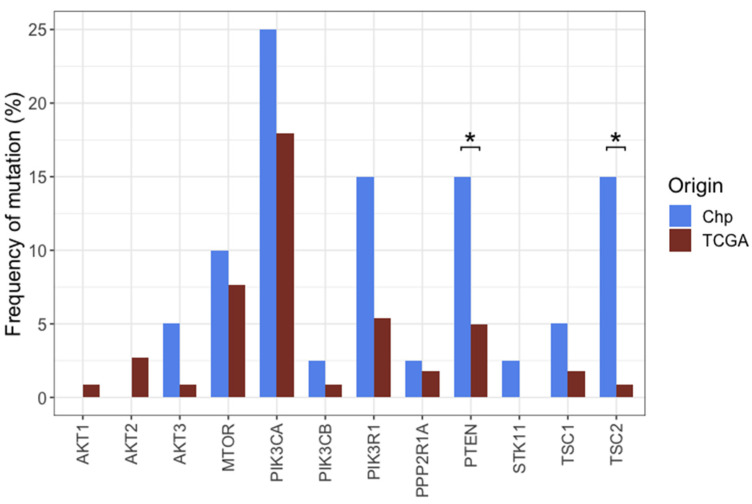
Mutation frequencies for the TCGA and Chp cohorts in the PI3K pathway genes. (* *p* < 0.05, Fisher’s exact test).

A gene-to-gene comparison was made to further investigate the difference between TCGA and Chp cohorts regarding the PI3K pathway. Significant differences were found in the frequencies of mutations in *TSC2* (*p* = 1.85 × 10^−5^) and *PTEN* (*p* = 0.042). Moreover, there is a general tendency for the Chp cohort to have a higher proportion of mutations in genes of this pathway, having a higher frequency in 10 out of the 12 genes analyzed (Figure 6). Additionally, although there was not a significant difference in the pairwise comparison between Chp and MSK-IMPACT, a gene-to-gene comparison showed a significant difference in *TSC2* mutation frequency (*p* = 3.062 × 10^−2^, Figure 4).

### 2.9. General Characteristics of the PI3K Pathway Altered Samples

**Figure 7:**
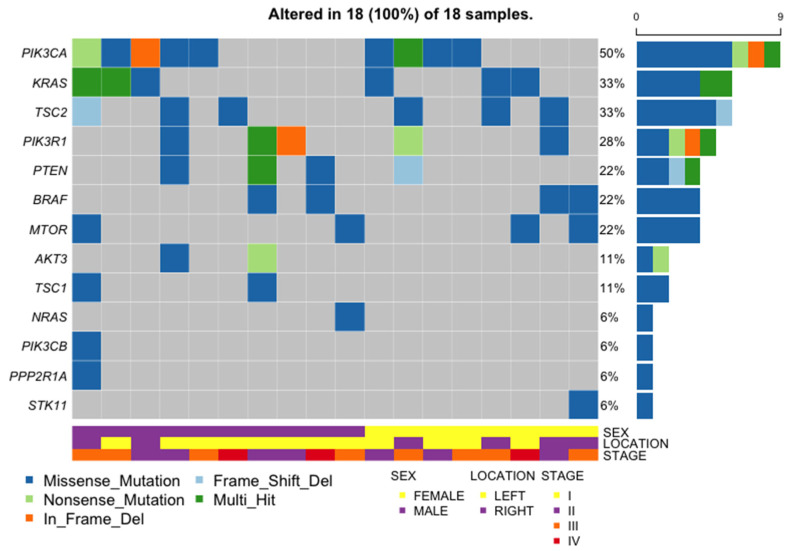
Distribution and type of mutations in the PI3K pathway and *KRAS*, *NRAS*, and *BRAF* genes, in samples enriched in modifications in the PI3K oncogenic pathway. The right panel shows the number of samples with mutations in the gene indicated in the same line on the left. The lower panel shows the sex of the patient and the location of the primary tumor.

Among the 18 samples with at least one of the PI3K pathway genes mutated, there was no significant difference between LCRC and RCRC (12/31 LCRC and 6/9 RCRC, *p*-value = 0.2534, Fisher’s exact test). Also, there was no difference between mutated and non-mutated samples either in LCRC or RCRC (*p*-values = 0.1269 and 0.3469, respectively, Fisher’s exact test). Men and women were affected equally (10/23 and 8/17, respectively, *p*-value = 1, Fisher’s exact test). The mean age at diagnosis was 65 years (range 25–86). Half of the samples have coexistent mutations in *KRAS*, *NRAS*, or *BRAF* (Figure 7).

## 3. Discussion

Somatic mutations and their frequencies in CRC have been assessed in many studies, and population-associated differences have been described. Indeed, some of the most frequently mutated genes in CRC have a wide range of variation in the mutation frequency according to ethnicity. For example, studies in patients of African and European ancestry have reported a higher prevalence of *KRAS* (60% vs. 50%) and *PIK3CA* (20% vs. 17%) mutations in African patients. Also, African patients have a lower prevalence of *BRAF* V600 mutations (2.0% vs. 6.0%) [28], and a Chinese cohort of 652 subjects showed frequencies of 23.7%, 25.8%, and 9.81% for *KRAS*, *TP53*, and *PIK3CA* [29]. Even further, dos Santos et al. found differences among the Brazilian population according to their ancestry; individuals with the highest proportion of African ascendancy had more frequent *NF1* and *BRAF* mutations, whereas those with the highest proportion of Native American ancestry had fewer *TP53* and *PIK3CA* mutations when compared to those of intermediate and the lowest proportion of Native American ancestry [30].

Knowing the frequency of specific mutations with diagnostic, therapeutic, and prognostic value is important to establish proper public health policies, for example, regarding treatment strategies and access.

### 3.1. Frequency of CRC Mutations in LATAM Cohorts

Unfortunately, detailed descriptions of mutation frequencies in the Latin American population are limited [31]. A study of 30 CRC Colombian patients reported frequencies of 13.3% in *KRAS* and 6.6% *TP53* mutations [32]. In a Chilean CRC cohort of 106 patients, mutation frequencies of 26%, 12%, and 18% for *KRAS*, *BRAF*, and *PI3KCA*, respectively, were reported [8]. In both studies, mutation frequencies for *KRAS*, *BRAF*, *PIK3CA*, and *TP53* were lower compared to the results obtained in this work. This difference can be explained by the methodology, since authors used qPCR and Sanger sequencing in these studies, limiting the analysis to specific codons or small regions. In fact, using NGS in a Brazilian cohort of 91 patients, dos Santos et al., found mutation frequencies similar to our results in *TP53* (56%), *BRAF* (8.8%), *FBXW7* (11%), and *PIK3CA* (15.4%) [30]. Nevertheless, they also found a higher incidence of *KRAS* mutations (52.7% vs. 30% in Chp) and a lower incidence of *ATM* mutations (6% vs. 20% in Chp). On the other hand, Takenaka et al. found different mutation frequencies in advanced rectal cancer from Brazilian and Argentinian patients in *TP53* (78.1% vs. 11%, respectively), *KRAS* (40.9% vs. 6%) and *FBXW7* (17.5% vs. 6%). It is important to mention that both dos Santos et al. and Takenaka et al. found *APC* as the most mutated gene in their cohorts [30,33]. Unfortunately, we do not know the mutation frequency in our cohort, since this gene was not analyzed.

### 3.2. Comparison of CRC Mutations with TCGA and MSK-IMPACT Cohorts

We also find differences in mutation frequencies when comparing Chp with other well-characterized cohorts, TCGA and MSK. Although information about the ethnicity of the patients in these cohorts was not completely available, they are mainly Caucasian [34,35]. The most notable finding was the higher frequency of *TSC2* and *PMS2* mutations in the Chilean cohort, compared to TCGA and MSK. *TSC2* mutations were also frequent in a Chinese cohort, and were associated with bad prognosis [36].

The six *TSC2* mutations found in six samples (1 per sample) were manually curated to discard any potential technical artifact (Figure 3). Five mutations were predicted deleterious using at least five prediction tools. These mutations have a COSMIC ID, with one entry in “large intestine”, and were classified as “somatic” according to a pipeline previously described [37]. Nevertheless, all six variants were looked for in large population germline variant databases ExAc and GnomAD (overall and population specific); ABraOM (Brazilian genomic variants); BIPMed (Brazilian Initiative on Precision Medicine); and in a local Chilean database. In all databases, these variants had a VAF mostly 0 or <0.0001. Nevertheless, one of these mutations with COSMIC ID was found with a VAF > 0.5 in the tumor, and thus a germline origin cannot be discarded in this case.

Regarding *PMS2* mutations, five mutations were found in five patients (one per sample), with VAF ranging from 0.05 to 0.48. Three mutations were found in LCRC and two in RCRC. Two of the mutations found in LCRC were classified as “novel”, c.T619C and c.460dupT.

### 3.3. Left vs. Right CRC

Regarding laterality, we observed previously described differences between LCRC and RCRC: LCRC was more frequent than RCRC; *KRAS*, *BRAF*, and *PIK3CA* were more frequently mutated in RCRC [6,38,39]; and *TP53* mutations were predominantly found in LCRC. These differences were also observed in the TCGA and MSK-IMPACT cohorts [39,40]. Given the small sample size (especially for RCRC), these differences were not significant in Chp. Nevertheless, a significant difference in the frequency of *TSC2* mutations was found between RCRC and LCRC, being higher in RCRC. As far as we know, this finding has not been reported before.

Although the MSK-IMPACT cohort has more significant differences between mutated genes on both sides, compared to TCGA, there is a tendency to agree between them. However, some differences could also be seen; for example, *FGFR1*, *FGFR2*, and *MSH2* were mainly mutated in RCRC in MSK-IMPACT, whereas the opposite was seen in TCGA. *TSC2* was more frequently mutated in RCRC in the MSK-IMPACT cohort, whereas in TCGA samples, only two mutations were found in LCRC and no mutations in RCRC.

*TSC1*/*TSC2* genes are part of the PI3K/AKT/mTOR pathway, which are the main negative regulators of mTOR activation. In the normal activation of the PI3K pathway, Akt phosphorylates *TSC1*/*TSC2*, allowing the activation of mTOR through the GTP-binding protein Rheb [41,42]. Mutations in *TSC1*/*TSC2* can lead to mTOR overactivation, promoting tumor growth [43]. The role of somatic mutations in *TSC2* is not described in CRC, but a study has shown that it could be associated with a worse prognosis when performing a combined prognostic model of mutations in five genes [36]. *TSC2* mutations or other PI3K components are supposed to sensitize tumors to mTOR inhibitors such as everolimus or sirolimus, which have been proposed as possible targeted therapies in other non-colorectal solid cancers [44,45,46,47]. Moreover, *TSC2* mutations may eventually affect EGFR-TKI response, since PI3K-AKT-mTOR pathway activation has been described as an important resistance mechanism in patients treated with EGFR TKIs (see below).

### 3.4. Comparison between Oncogenic Pathways

Hyperactivation of the PI3K/AKT/mTOR pathway may be an opportunity for treatment with mTOR inhibitor therapy, which has been suggested to have a beneficial effect when not associated with *KRAS* mutations [48]. Previous studies have found greater activation of this PI3K pathway in populations of African ancestry compared to populations of European ancestry [28]. On the other hand, a similar frequency of activation of the PI3K pathway was found in a Brazilian cohort to those we observed in TCGA (23.1% and 25.56%, respectively) [30]. Here, we found greater activation of the PI3K pathway compared to the TCGA cohort (45% in Chp vs. 25.56%). This is caused not only by the significant increase in *TSC2* mutations, but also *PIK3R1* and *PTEN*, which showed a higher frequency of mutations in Chp compared to TCGA (15% vs. 5.38%, *p*-value = 0.06 and 15% vs. 4.93%, *p*-value = 0.042).

As mentioned above, in addition to response to mTOR inhibitors, the oncogenic activation of the PI3K/AKT/mTOR pathway members have been involved in the resistance to EGFR/BRAF inhibitors. For instance, a PIK3R1 mutation emerged in a patient with a *BRAF* V600E mutation after dual treatment with cetuximab and vemurafenib, suggesting a possible role in acquired resistance to this therapy [20], whilst *PIK3CA* and *PTEN* mutations became detectable in circulating tumor DNA from metastatic CRC patients after treatment with panitumumab [49]. Colon cancer cell lines showed increased resistance to cetuximab when *PTEN* expression is lost or *PIK3CA* is mutated, and an even higher degree of resistance when any of these alterations is concurrently present with *RAS*/*BRAF* mutations [50]. Additionally, *PTEN* loss of expression has been associated with resistance to cetuximab in metastatic CRC [51,52]. Thus, it is plausible to expect that *PTEN* loss-of-function variants might influence the performance of a patient to anti-EGFR therapy.

We also observed a slightly higher, but not significant, mutation frequency in *PIK3CA* (17.94% TCGA vs. 25% Chp, *p*-value = 0.4069). Mutations in this gene have been previously associated with resistance to first-line chemotherapy, poor prognosis [17], and resistance to EGFR-targeted therapy [52]. However, this latter point could be controversial, as the predictive value of PIK3CA mutations might be restricted to those affecting exon 20 [18].

Genetic background likely explains some of the previously observed differences between Latin Americans, and specifically between Chileans and international cohorts. In Chile, the average individual possesses roughly 48% Native American ancestry with less than 3% African ancestry, significantly lower than populations like Brazilians and Colombians [34]. Furthermore, Chilean European ancestry (around 50%) shows greater similarity to Spaniards and Italians, who are also underrepresented in cohorts like TCGA and MSK [35,53]. While this study only captured self-reported ethnicity data from 17 patients, most identified as “Chilean” (admixed), and all originated from a region with a predominantly admixed population, reflecting the average genetic makeup of Chile. Future studies with larger sample sizes and combined genetic ancestry and self-reported ethnicity data would provide a more comprehensive picture and enable subgroup analysis based on ethnicity.

A population’s specific genetic composition, interacting with its geographical and/or cultural environment, may influence the activation of certain carcinogenic pathways, leading to variations in tumor mutational profiles. For instance, some genetic loci exhibit highly divergent allele frequencies across diverse geographic regions or ancestries. One such locus in Chileans displays significant allele frequency differentiation within the Low-density lipoprotein Receptor Related Protein 1B (*LRP1B*) gene, previously linked to obesity, a known CRC risk factor [54,55]. LRP1B functions as a tumor suppressor, regulating the extracellular environment to limit cancer cell invasion [56]. Notably, recent research also suggests LRP1B regulates the PI3K/AKT pathway in Hepatocellular Carcinoma (HCC) [57]. Therefore, polymorphisms in this gene, combined with high-fat diets, could potentially explain the rising obesity and CRC rates in Chile, along with the higher prevalence of PI3K pathway mutations described in this work.

## 4. Materials and Methods

### 4.1. Samples and Sequencing

Formalin-fixed paraffin-embedded (FFPE) tumor samples were obtained from forty Chilean CRC patients (Chp) treated at the University Hospital and collected through the “Biobanco de Tejidos de la Universidad de Chile, BTUCH”. DNA was isolated using RecoverAll™ Total Nucleic Acid Isolation Kit (ThermoFisher, Waltham, MA, USA). In total, 20ng of DNA were used to library preparation using the Oncomine™ Comprehensive Assay V3 (Thermo Fisher Scientific) following product instructions. Sequencing was performed in an Ion S5™ Sytem using Ion 550 kit-CHEF (Thermo Fisher Scientific).

### 4.2. Bioinformatic Analysis, Variant Calling and Classification

The preprocessing and data processing were carried out using the Oncomine Comprehensive Assay v3 (OCA) v5.18 DNA workflow, using default parameters and the hg19 genome reference. For alignment and variant calling, stringent parameters were defined. Single Nucleotide Variants (SNV) required a minimum allele frequency of 5%, while Indels required 7%. The minimum coverage for a variant to be considered was set at 10× for SNV and Indels. Additionally, the minimum coverage for the variant location was set at 50×.

Variant annotation was performed using ANNOVAR [58] including RefGene, GnomAD v2.1.1, ESP6500, ExAC v0.3, 1000 Genomes phase 3, CADD v1.3, dbSNP v150, COSMIC v94, CLINVAR 2021, ICGC28, ABraOM, and Snp138NonFlaged. To enhance the filtering of germline variants in tumor samples, large and local population germline variant databases were interrogated: ExAc and GnomAD (overall and population specific); BIPMed (Brazilian Initiative on Precision Medicine); and a Chilean database composed by variants imputed from genotyping 2 arrays with 1313 and 2249 samples from Chilean individuals [59,60], and whole exome sequencing (WES) data from 87 individuals [61].

### 4.3. Public Databases

Mutation data of CRC samples were extracted from The Cancer Atlas Genome firehose legacy and Memorial Sloan Kettering Clinical Sequencing cohorts [56] (hereafter TCGA and MSK-IMPACT, respectively) through cBioportal (https://www.cbioportal.org, accessed on 25 march 2022). Only somatic protein-affecting variants found in primary tumors were analyzed in both datasets. The sample size for the TCGA and MSK-IMPACT cohorts were 223 and 514, respectively.

### 4.4. Gene Selection

The OncoKB database (https://www.oncokb.org, accessed on 7 July 2022) was consulted to select genes for further comparisons between different cohort data sets. To do that, filtering based on colorectal cancer was done, choosing all the genes affected by alterations other than those resulting from large structural alterations (amplifications, translocations, etc.) and including those valid both for colorectal cancer and for “all solid tumors”, disregarding the level of evidence. These genes were: *BRAF*, *KRAS*, *NRAS*, *NTRK1*, *NTRK3*, *ARID1A*, *CDK12*, *CDKN2A*, *FGFR1*, *FGFR2*, *FGFR3*, *MTOR*, *NF1*, and *PTEN*. Although specific alterations were associated with drugs in some cases, here we compared all mutations affecting those genes in all cohorts.

Other genes classically related to CRC or with potential interest were also considered. Those genes were: *BRCA1*, *BRCA2*, *MLH1*, *MSH2*, *MSH6*, *PMS2*, *POLE*, *RB1*, *TP53*, *TSC1,* and *TSC2*.

### 4.5. Pathways Analyses

Pathways analyses were made using the OncogenicPathways tool from the Maftool package, separately considering the RTK-RAS and PI3K pathways. Given that MSK-IMPACT, TCGA, and Chp cohorts were sequenced using different approaches, only mutations in shared gene regions were performed.

### 4.6. Statistical Data Analysis

Differences in age at diagnosis between right and left CRC were done using the Wilcoxon test. Fisher’s exact test was used to compare mutation frequencies within the Chp cohort (either when comparing RCRC and LCRC, men and women, etc.). The Chi-square test was performed for comparing mutation frequencies among all three cohorts. *p*-values were adjusted according to the Benjamini–Hochberg’s method [62], and pairwise Chi-square test with Yates’ continuity correction was used for post hoc comparison in case of a significant *p*-value (<0.05). All statistical and data analyses were done using R 4.1.1 software. Grouped bar plots, oncoplots, and pathway plots were generated using ggplot2 and MafTools packages in R 4.1.1 software.

## 5. Conclusions

In this preliminary work, we found differences in *TSC2* and *PMS2* mutation frequency and PI3K oncogenic pathway activation in a cohort of 40 CRC from Chilean patients compared to other cohorts. These differences could be related to ethnicity and genetic background. However, given that the small size of this study does not allow further stratification of patients and tumors, intrinsic disease-related factors could also contribute to differences [53,54].

Nevertheless, these findings may have a high impact on clinical decisions at individual and public health levels. For instance, our study group showed lower frequency of mutations in the TRK/RAS pathway than international cohorts, suggesting that Chilean patients may benefit from anti-EGFR therapies. However, on the other side, the most frequently mutated genes/pathways are associated with resistance to these medications.

Further exploration in larger follow-up studies with a significantly larger sample size and complete demographic and genetic information are needed to validate these results and to definitively assess TSC2 mutation frequency, its association with tumor location, and the influence of genetic background and other factors on the oncogenic process.

These findings highlight the urgent need of tumor characterization of all under-represented populations and increase diversity in late-stage clinical trials.

## Figures and Tables

**Table 1:** General characteristics of the patients and their tumors.

Characteristic		RCRC	LCRC	TOTAL	*p*-Value (LCRC vs. RCRC) *
Cases		9	31	40	**0.000001508**
Gender	Males	3	20	23	0.1338
	Females	6	11	17	0.1338
Mean age at diagnosis		71.22 (range 51–86) years	61.35 (range 25–82) years	63.58 (range 25–86) years	0.06236
Stage	I	0	2	2	1
	II	4	12	16	1
	III	4	13	17	1
	IV	1	4	5	1
Histological diagnosis	Tubular adenocarcinoma	3	15	18	0.4761
	Adenocarcinoma, NOS	3	10	13	N/A
	Papillary-tubular adenocarcinoma	1	1	2	0.4038
	Poorly differentiated with signet-cells carcinoma	0	1	1	1
	Mucinous adenocarcinoma	1	0	1	0.225
	Adenocarcinoma mixed tubular and mucinous	0	1	1	1
	Not specified	1	3	4	N/A
Ethnicity	Chilean	2	9	11	1
	Mapuche	2	3	5	0.2677
	European	0	1	1	1
	Not specified	6	17	23	N/A

* Statistically significant differences are highlighted in bold. Fisher’s exact test.

**Table 2:** Comparison between LCRC and RCRC in MSK-IMPACT and TCGA, with *p*-values determined using a chi-square test. Statistically significant *p*-values (adjusted for false discovery rate) are highlighted in bold.

	MSK-IMPACT				TCGA			
Gene	LCRC (n = 311)	RCRC (n = 199)	*p*-Value	adj *p*-Value	LCRC (n = 142)	RCRC (n = 78)	*p*-Value	adj *p*-Value
*ARID1A*	11.58% (36)	24.12% (48)	**3.140 × 10^−4^**	**1.13 × 10^−3^**	6.34% (9)	16.67% (13)	**2.725 × 10^−2^**	1.18 × 10^−1^
*BRAF*	8.36% (26)	23.62% (47)	**3.015 × 10^−6^**	**3.92 × 10^−5^**	2.82% (4)	23.08% (18)	**5.191 × 10^−6^**	**1.35 × 10^−4^**
*BRCA1*	4.5% (14)	6.03% (12)	5.76 × 10^−1^	5.99 × 10^−1^	2.11% (3)	385% (3)	7.471 × 10^−1^	9.42 × 10^−1^
*BRCA2*	6.75% (21)	22.11% (44)	**7.924 × 10^−7^**	**2.06 × 10^−5^**	8.45% (12)	12.82% (10)	4.245 × 10^−1^	8.49 × 10^−1^
*CDK12*	2.89% (9)	7.04% (14)	**4.774 × 10^−2^**	6.53 × 10^−2^	2.82% (4)	6.41% (5)	3.516 × 10^−1^	7.62 × 10^−1^
*CDKN2A*	1.93% (6)	4.02% (8)	2.577 × 10^−1^	2.91 × 10^−1^	0% (0)	1.28% (1)	7.606 × 10^−1^	9.42 × 10^−1^
*FGFR1*	1.93% (6)	4.52% (9)	1.550 × 10^−1^	1.83 × 10^−1^	2.11% (3)	1.28% (1)	1 × 10^0^	1 × 10^−0^
*FGFR2*	2.25% (7)	5.03% (10)	1.471 × 10^−1^	1.82 × 10^−1^	3.52% (5)	1.28% (1)	5.873 × 10^−1^	9.42 × 10^−1^
*FGFR3*	1.29% (4)	6.53% (13)	**3.009 × 10^−3^**	**6.52 × 10^−3^**	0% (0)	2.56% (2)	2.402 × 10^−1^	5.68 × 10^−1^
*KRAS*	41.16% (128)	57.79% (115)	**3.470 × 10^−4^**	**1.13 × 10^−3^**	41.55% (59)	46.15% (36)	6.049 × 10^−1^	9.42 × 10^−1^
*MLH1*	1.93% (6)	5.53% (11)	5.053 × 10^−2^	6.57 × 10^−2^	2.82% (4)	5.13% (4)	6.173 × 10^−1^	9.42 × 10^−1^
*MSH2*	2.57% (8)	8.04% (16)	**8.538 × 10^−3^**	**1.59 × 10^−2^**	3.52% (5)	2.56% (2)	1 × 10^0^	1 × 10^−0^
*MSH6*	3.54% (11)	11.06% (22)	**1.462 × 10^−3^**	**3.8 × 10^−3^**	4.23% (6)	11.54% (9)	7.523 × 10^−2^	2.79 × 10^−1^
*MTOR*	3.54% (11)	14.07% (28)	**2.722 × 10^−5^**	**1.42 × 10^−4^**	5.63% (8)	11.54% (9)	1.919 × 10^−1^	5.68 × 10^−1^
*NF1*	4.18% (13)	13.07% (26)	**4.442 × 10^−4^**	**1.28 × 10^−3^**	4.23% (6)	6.41% (5)	6.98 × 10^−1^	9.42 × 10^−1^
*NRAS*	4.18% (13)	5.03% (10)	8.182 × 10^−1^	8.18 × 10^−1^	9.15% (13)	8.97% (7)	1 × 10^0^	1 × 10^−0^
*NTRK1*	2.57% (8)	8.54% (17)	**4.569 × 10^−3^**	**9.14 × 10^−3^**	2.11% (3)	3.85% (3)	7.471 × 10^−1^	9.42 × 10^−1^
*NTRK3*	2.25% (7)	6.53% (13)	**2.808 × 10^−2^**	**4.06 × 10^−2^**	2.11% (3)	11.54% (9)	**8.419 × 10^−3^**	5.47 × 10^−2^
*PIK3CA*	19.61% (61)	38.19% (76)	**6.347 × 10^−6^**	**5.16 × 10^−5^**	11.27% (16)	30.77% (24)	**6.618 × 10^−4^**	**5.74 × 10^−3^**
*PMS2*	0.96% (3)	2.51% (5)	3.139 × 10^−1^	3.4 × 10^−1^	1.41% (2)	5.13% (4)	2.349 × 10^−1^	5.68 × 10^−1^
*POLE*	6.75% (21)	14.07% (28)	**9.839 × 10^−3^**	**1.71 × 10^−2^**	5.63% (8)	16.67% (13)	**1.534 × 10^−2^**	7.98 × 10^−2^
*PTEN*	7.07% (22)	16.08% (32)	**2.091 × 10^−3^**	**4.94 × 10^−3^**	4.93% (7)	5.13% (4)	1 × 10^0^	1 × 10^−0^
*RB1*	3.54% (11)	8.54% (17)	**2.631 × 10^−2^**	**4.96 × 10^−2^**	2.11% (3)	6.41% (5)	2.104 × 10^−1^	5.68 × 10^−1^
*TP53*	81.03% (252)	62.81% (125)	**7.944 × 10^−6^**	**5.16 × 10^−5^**	65.49% (93)	35.9% (28)	**4.515 × 10^−5^**	**5.87 × 10^−4^**
*TSC1*	1.93% (6)	6.03% (12)	**2.765 × 10^−2^**	**4.06 × 10^−2^**	1.41% (2)	2.56% (2)	9.312 × 10^−1^	1 × 10^−0^
*TSC2*	1.93% (6)	10.55% (21)	**5.35 × 10^−5^**	**2.32 × 10^−4^**	1.41% (2)	0% (0)	7.562 × 10^−1^	9.42 × 10^−1^

## Data Availability

TCGA data were downloaded from https://www.cbioportal.org/study/summary?id=coadread_tcga, and MSK-IMPACT was downloaded from https://www.cbioportal.org/study/summary?id=msk_impact_2017, both accessed on 25 March 2022. Data generated during this study will be available upon request.

## Supplementary Materials

The following supporting information can be downloaded at: https://www.mdpi.com/article/10.3390/ijms25094695/s1.
