# Supplementary material for: Distinct Driver Pathway Enrichments and a High Prevalence of TSC2 Mutations in Right Colon Cancer in Chile: A Preliminary Comparative Analysis
Source: Int J Mol Sci. 2024 Apr 25;25(9):4695. doi: 10.3390/ijms25094695 (PMC11083322; doi:10.3390/ijms25094695)
Supplement: Supplementary file 1 [file ijms-25-04695-s001.zip › ijms-2890573-supplementary.pdf]

Table S1. Somatic mutations identified in actionable genes in Chilean patients diagnosed with colorectal cancer (CRC)

Gene	Frequency (N° of samples with the mutation)	ntChange (c.DNA)	aaChange (p.)
KRAS	1	T68G	L23R
	1	G38A	G13D
	1	G35C	G12A
	1	G34T	G12C
	2	G35A	G12D
	6	G35T	G12V
	1	G175A	A59T
	1	G274T	D92Y
NRAS	1	G178A	G60R
BRAF	1	C1750A	L584I
	1	G1405C	G469R
	3	T1799A	V600E
PMS2	1	G1640T	C547F
	1	G1585A	E529K
	1	T619C	Y207H
	1	C1553A	S518Y
	1	460dupT	S154fs
MSH2	1	C1622T	T541I
	1	G680T	R227I
	1	A2495G	E832G
	3	G1738T	E580X
MSH6	1	T380C	V127A
	1	G280T	E94X
	1	G1055A	R352Q
	1	G475T	A159S
	1	G173A	R58K
PIK3CA	2	G1633A	E545K
	1	G1030A	V344M
	1	G1624A	E542K
	1	335_337del	112_113del
	1	A1637G	Q546R
	1	G241A	E81K
	1	G278A	R93Q
	1	C3067T	R1023X
	1	A3140G	H1047R
TSC1	1	G2757T	E919D
	1	G530A	R177Q
TSC2	1	C4453T	R1485C
	1	C3857T	S1286L
	1	G496A	E166K
	1	C3619T	R1207C
	1	G233A	R78H
	1	1359_1360del	R453fs
PTEN	1	G895T	E299X
	1	T299C	L100P
	1	G389A	R130Q
	1	963delA	T321fs
	1	G21T	E7D
	1	G395A	G132D
MTOR	2	C6721T	P2241S
	1	A7498T	I2500F
	1	G4291A	G1431R

Table S2. Comparison among Chp, MSK-IMPACT and TCGA cohorts.

Gene	Chp (n = 40)	TCGA (n = 223)	MSK-IMPACT (n = 514)	*p-value*	adj *p-value*
TP53	45% (18)	54.71% (122)	73.74% (379)	**1.698 x 10-6**	0.000044148
PMS2	12.5% (5)	2.69% (6)	1.56% (8)	**8.651 x 10-5**	0.001124630
TSC2	15% (6)	0.9% (2)	5.25% (27)	**1.463 x 10-4**	0.001267933
PIK3CA	22.5% (9)	1.79% (40)	27.43% (141)	**2.153 x 10-2**	0.139945000
NRAS	2.5% (1)	8.97% (20)	4.47% (23)	**3.563 x 10-2**	0.180050000
PTEN	10% (4)	4.93% (11)	10.7% (55)	**4.155 x 10-2**	0.180050000
ARID1A	17.5% (7)	9.87% (22)	16.34% (84)	6.246 x 10-2	0.219830000
KRAS	30% (12)	43.05% (96)	47.67% (245)	6.764 x 10-2	0.219830000
CDK12	10% (4)	2.69% (6)	4.47% (23)	9.782 x 10-2	0.256022000
POLE	20% (8)	9.42% (21)	9.53% (49)	9.847 x 10-2	0.256022000
CDKN2A	2.5% (1)	0.45% (1)	2.72% (14)	1.331 x 10-1	0.314600000
MSH2	10 % (4)	3.14% (7)	44.44 (24)	1.490 x 10-1	0.314600000
FGFR3	2.5% (1)	0.9% (2)	3.31% (17)	1.649 x 10-1	0.314600000
NF1	12.5% (5)	4.93% (11)	7.59% (39)	1.694 x 10-1	0.314600000
BRCA1	7.5% (3)	2.69% (6)	5.06% (26)	2.338 x 10-1	0.399329412
NTRK1	7.5% (3)	2.69% (6)	4.86% (25)	2.542 x 10-1	0.399329412
NTRK3	0% (0)	5.38% (12)	3.89% (20)	2.611 x 10-1	0.399329412
BRAF	12.5% (5)	10.31% (23)	14.2% (73)	3.519 x 10-1	0.495778947
TSC1	5% (2)	18.18% (4)	3.5% (18)	3.623 x 10-1	0.495778947
FGFR1	0% (0)	18.18% (4)	2.92% (15)	3.903 x 10-1	0.507390000
RB1	7.5% (3)	3.59% (8)	5.45% (28)	4.332 x 10-1	0.536342857
FGFR2	0% (0)	2.69% (6)	3.31% (17)	4.742 x 10-1	0.549956522
MLH1	0% (0)	3.59% (8)	3.31% (17)	4.865 x 10-1	0.549956522
BRCA2	12.5% (5)	9.87% (22)	12.65% (65)	5.573 x 10-1	0.603741667
MTOR	10% (4)	7.62% (17)	7.59% (39)	8.575 x 10-1	0.891800000
MSH6	7.5% (3)	6.73% (15)	6.42% (33)	9.588 x 10-1	0.958800000

**p-value according to chi-square test. Significant values are in bold.*
